# Clinical outcome after surgery for lumbar spinal stenosis in patients with insignificant lower extremity pain. A prospective cohort study from the Norwegian registry for spine surgery

**DOI:** 10.1186/s12891-019-2407-5

**Published:** 2019-01-22

**Authors:** Erland Hermansen, Tor Åge Myklebust, Ivar Magne Austevoll, Frode Rekeland, Tore Solberg, Kjersti Storheim, Oliver Grundnes, Jørn Aaen, Jens Ivar Brox, Christian Hellum, Kari Indrekvam

**Affiliations:** 1 Department of Orthopedic Surgery, Ålesund Hospital, Møre and Romsdal Hospital Trust, Ålesund, Norway; 2 Kysthospitalet in Hagevik, Orthopedic Clinic, Haukeland University Hospital, Bergen, Norway; 3 Department of Clinical Medicine, University of Bergen, Bergen, Norway; 4 Department of Research, Møre og Romsdal Hospital Trust, Ålesund, Norway; 5 Department of Neurosurgery, University Hospital of Northern Norway, Tromsø, Norway; 6 Department of Clinical Medicine, University of Tromsø The Arctic University of Norway, Tromsø, Norway; 7 University Hospital of North, Norwegian National Registry for spine surgery, Tromsø, Norway; 8 Communication and Research Unit for Musculoskeletal Disorders (FORMI), Oslo University Hospital and University of Oslo, Oslo, Norway; 9 Department of Orthopedics, Akershus University Hospital, Oslo, Norway; 10 Department of Physical Medicine and Rehabilitation, University of Oslo, Oslo, Norway; 11 Department of Orthopedics, Oslo University Hospital, Oslo, Norway

**Keywords:** Lumbar spinal stenosis, Lower extremity pain, Register trial, Clinical outcome

## Abstract

**Background:**

Spinal stenosis is a clinical diagnosis in which the main symptom is pain radiating to the lower extremities, or neurogenic claudication. Radiological spinal stenosis is commonly observed in the population and it is debated whether patients with no lower extremity pain should be labelled as having spinal stenosis. However, these patients is found in the Norwegian Registry for Spine Surgery, the main object of the present study was to compare the clinical outcomes after decompressive surgery in patients with insignificant lower extremity pain, with those with more severe pain.

**Methods:**

This study is based on data from the Norwegian Registry for Spine Surgery (NORspine). Patients who had decompressive surgery in the period from 7/1–2007 to 11/3–2013 at 31 hospitals were included. The patients was divided into four groups based on preoperative Numeric Rating Scale (NRS)-score for lower extremity pain. Patients in group 1 had insignificant pain, group 2 had mild or moderate pain, group 3 severe pain and group 4 extremely severe pain. The primary outcome was change in the Oswestry Disability Index (ODI). Successfully treated patients were defined as patients reporting at least 30% reduction of baseline ODI, and the number of successfully treated patients in each group were recorded.

**Results:**

In total, 3181 patients were eligible; 154 patients in group 1; 753 in group 2; 1766 in group 3; and 528 in group 4. Group 1 had significantly less improvement from baseline in all the clinical scores 12 months after surgery compared to the other groups. However, with a mean reduction of 8 ODI points and 56% of patients showing a reduction of at least 30% in their ODI score, the proportion of patients defined as successfully treated in group 1, was not significantly different from that of other groups.

**Conclusion:**

This national register study shows that patients with insignificant lower extremity pain had less improvement in primary and secondary outcome parameters from baseline to follow-up compared to patients with more severe lower extremity pain.

## Background

Radicular pain in the lower extremities known as neurogenic claudication is considered to be the main symptom of Lumbar Spinal Stenosis (LSS) [1–3]. According to criteria from the North American Spine Society, the most dominant historical and physical finding in lumbar spinal stenosis, is gluteal or lower extremity pain, which is exacerbated by walking and relieved by flexion of the spine [4]. Whether or not patients have this symptom is considered one of the most important clinical signs, when evaluating a patient for lumbar spinal stenosis [5]. However, most of the patients with symptoms of lumbar spinal stenosis describe both leg pain and low back pain [6], and it may be difficult for patients to define whether the leg pain or back pain is dominant [7].

Low back pain is multifactorial, and several explanations are possible. Radiological findings of lumbar spinal stenosis may be incidental, since a high proportion of radiological lumbar spinal stenosis has been documented in asymptomatic subjects [8, 9]. Dominance of leg pain is commonly considered to be best indication for decompressive surgery. In most trials the patients report postoperative improvement in functional scores, leg pain and low back pain, after posterior decompression [10, 11]. Some studies have tried to identify predictors of clinical outcomes after posterior decompression for lumbar spinal stenosis [12], but very few strong predictors has been found [13]. Surgical treatment for lumbar spinal stenosis is considered by many to be superior to non-surgical treatment [14–17], but the most recent Cochrane review of the efficacy of surgical treatment versus non-surgical treatment, in which only trials with neurogenic claudication as main inclusion criterion were included, did not support this conclusion [18]. The effect of surgery in patients with atypical symptoms and radiologically verified lumbar spinal stenosis is to our knowledge not sufficiently researched.

The aim of the present study was to investigate whether or not the degree of preoperative lower extremity pain influences the clinical results after decompressive surgery for lumbar spinal stenosis.

## Methods

### Study population

This cohort study is based on data from the Norwegian Registry for Spine Surgery (NORspine). Patients labeled as having had surgery for lumbar spinal stenosis with midline retaining decompression in the period from 7/1–2007 to 11/3–2013 were included. In this period 31 of the Norwegian hospitals (86%) reported to the register. All patients receive oral and written information among their participation in the registry. They sign a written consent to participate in the registry. The registry protocol was approved by the Norwegian board of ethics, REC Central (2014/98).

**Fig. 1:**
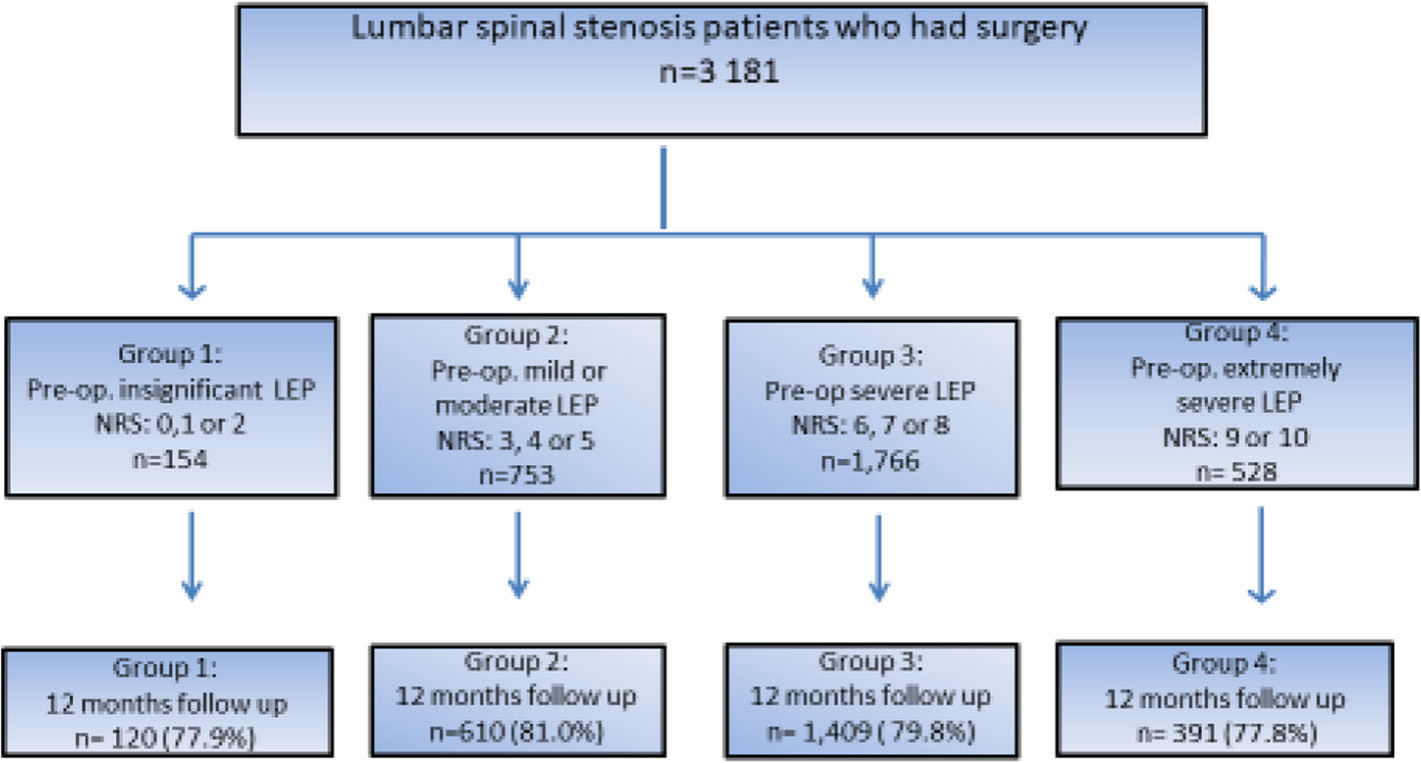
Chart showing the grouping and follow-up of patients with lumbar spinal stenosis who underwent decompression surgery; information obtained from the Norwegian Registry for Spine Surgery (NORspine). LEP: Lower extremity pain

The patients in this study were divided into four groups based on patient-reported preoperative Numeric Rating Scale (NRS) – a score for lower extremity pain. Patients in group 1 had insignificant lower extremity pain (NRS-score = 0, 1 and 2), group 2 had mild or moderate pain (NRS-score = 3, 4 and 5), group 3 had severe pain (NRS-score = 6, 7 and 8) and group 4 had extreme severe pain (NRS-score = 9 and 10) (Fig. 1).

### Patient reported outcome measures

The NORspine uses a recommended [19–24] set of patient reported outcome measures (PROMs). The questionnaires are self-administered at admission for surgery (baseline) and at 3 and 12 months follow-up. At baseline the forms also include questions about demographics and lifestyle issues.

During the hospital stay the surgeon records data concerning exact spinal diagnosis for surgery, possible spinal co-diagnosis, comorbidity, radiological classifications, the American Society of Anesthesiologists (ASA) grade, perioperative complications, operation method, duration of surgery and hospital stay. Patients were selected for the present study if the surgeon had ticked the registration form for the diagnosis lumbar spinal stenosis (without any additional spinal co-diagnosis, as degenerative spondylolisthesis) and that midline retaining decompression had been performed (without additional fusion).

### Outcome assessment

The primary outcome was change in pain-related physical function, assessed by the Norwegian version of the Oswestry Disability Index (ODI) questionnaire, version 2.0 [20]. It contains ten questions related to pain limitations in activities of daily living, ranging from 0 (no disability) to 100 (worst possible). Successfully treated patients were defined as patients reaching at least 30% reduction of the baseline ODI score [25], and the number of patients in each group reaching this level was recorded. These analyses were performed with and without adjusting for baseline values.

### Secondary outcomes

Secondary outcome measures were changes in NRS (from 0 (none) to 10 (worst possible)) for leg and back pain, and health related quality of life measured by the EQ-5D (ranging from − 0.59 to 1).

The ODI, NRS pain scales, and EQ-5D have shown good validity and reliability, and the Norwegian versions of these instruments have shown good psychometric properties [22–24].

### Statistics

Descriptive statistics for baseline characteristics were performed, as well as for clinical outcomes. Frequencies were used for categorical variables, whereas mean and standard deviations were used for continuous variables. To assess differences in distributions across the four patient groups, the standard Chi-square test was used for categorical variables, whereas ANOVA tests were used for continuous variables. Standard t-tests were used to analyze clinical outcomes separately for each patient group. Since differences in baseline parameters between the four groups were found, multivariate linear and logistic regressions were used to further analyze the association between lower extremity pain and clinical outcomes. The variables in the linear and logistic regressions were age, sex, Body Mass Index, smoking status, preoperative ODI, preoperative NRS-score for leg pain and preoperative NRS-score for low back pain. Age, Body Mass Index, preoperative ODI and EQ-5D were included as linear variables and smoking status, sex and the NRS-scores were categorical variables. When analyzing the probability to be classified as successfully treated logistic regression were used, adjusting for baseline values.

## Results

### Baseline characteristics

There were 3181 patients with lumbar spinal stenosis who underwent decompressive surgery in this register cohort. None of these patients had spinal co diagnosis. Regarding preoperative pain in the lower extremities, there were 154 patients in group 1; 753 patients in group 2; 1766 patients in group 3; and 528 patients in group 4 (Fig. 1).

The follow up response rate after 12 months was around 80% (77.8–81.0%) (Fig. 1). Baseline data are presented in Table 1. Statistically significant differences in baseline data across the four groups were found in all pain and function parameters. There were statistically significant differences between the four groups in age, sex and in smoking, but not in BMI. Patients in group 1 were younger, and there were higher proportions of males and non-smokers compared to the other groups. At baseline we found that the higher the NRS-score for leg pain, the higher were the scores for ODI, NRS-score for low back pain, and EQ-5D (*p* < 0.05).

**Table 1:** Baseline demographic data for patients with lumbar spinal stenosis undergoing decompressive surgery. The patients are divided into four groups according to their preoperative Numeric Rating Scale (NRS) –score for lower extremity pain

	Group 1 NRS = 0–2 *n* = 154	Group 2 NRS = 3–5 *n* = 753	Group 3 NRS = 6–8 *n* = 1766	Group 4 NRS = 9–10 *n* = 528	*p*-value
Age Mean (SD)	62.5 (12.0)	63.0 (11.5)	62.8 (11.0)	64.8 (12.0)	*p* < 0.05_a_
Sex % men	62.3	61.2	49.6	30.9	*p* < 0.05_b_
Smoke %	22.2	23.7	27.7	29.8	*p* < 0.05_b_
BMI Mean (SD)	27.3 (4.2)	27.2 (4.2)	27.3 (4.3)	26.8 (4.4)	*p* = 0.27_a_
ODI-score Mean (SD)	25.9 (15.2)	31.3 (12.3)	39.3 (13.2)	52.5 (15.1)	*p* < 0.05_a_
Back-pain (NRS) Mean (SD)	3.6 (2.7)	4.8 (1.8)	6.6 (1.7)	8.5 (1.9)	*p* < 0.05_a_
EQ-5D-score Mean (SD)	0.60 (0.28)	0.55 (0.25)	0.36 (0.31)	0.12 (0.26)	*p* < 0.05_a_
Number of levels Mean (SD)	1.4 (0.7)	1.3 (0.6)	1.8 (0.6)	1.3 (0.5)	*p* = 0.07_a_

a = ANOVA-test. B = Pearsons Chi-test

### Primary outcome

**Fig. 2:**
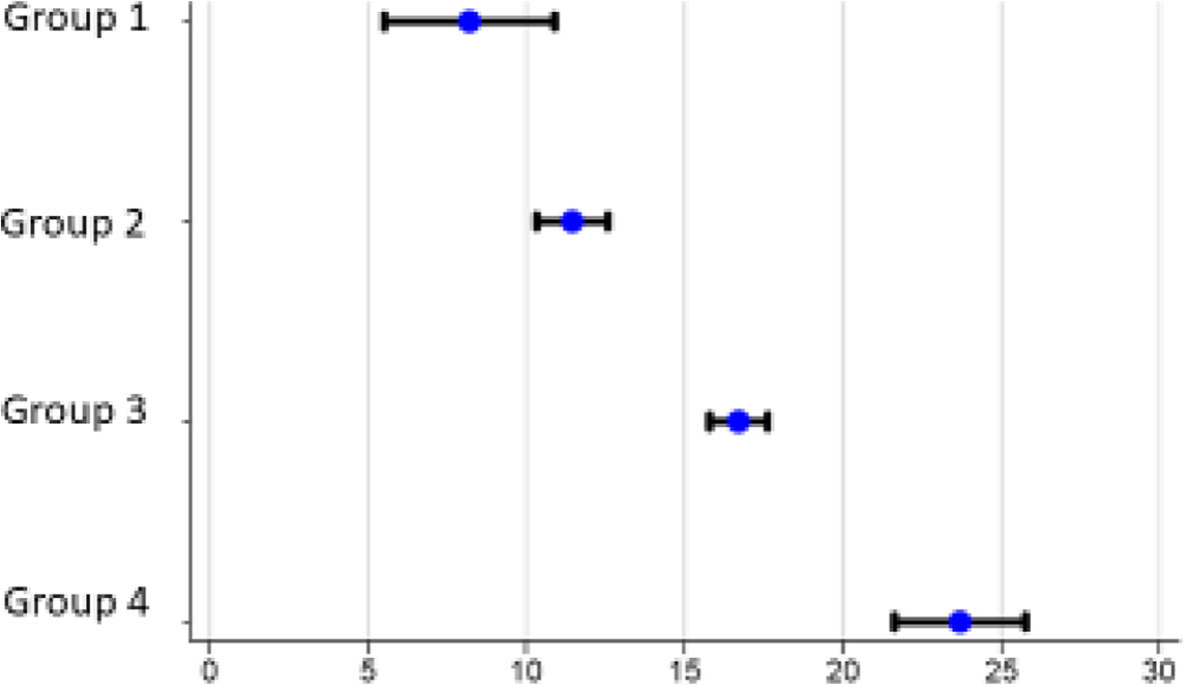
Mean change in ODI score. Mean change, decrease in ODI (with SD) from baseline to 12 months postoperative follow up. Exact values given in Table 2. The patients are divided into four groups according to their preoperative Numeric Rating Scale (NRS) –score for lower extremity pain. Group 1 = NRS-score 0, 1 and 2, group 2 = NRS-score 3, 4 and 5, group 3 = NRS-score 6, 7 and 8 and group 4 = NRS-score 9 and 10 for lower extremity pain

The mean change in ODI from baseline to 12 months postoperatively was significantly different between the four groups (Fig. 2 and Table 2).

**Table 2:** Change in patient reported outcomes from baseline to 12 months after surgery. Postive values indicate clinical improvement, negative values indicate a worsening. The patients are divided into four groups according to their preoperative Numeric Rating Scale (NRS) –score for lower extremity pain

Improvement from baseline after 12 months follow up	Group 1 NRS = 0–2 *n* = 154	Group 2 NRS = 3–5 *n* = 753	Group 3 NRS = 6–8 *n* = 1766	Group 4 NRS = 9–10 *n* = 528	*p*-value
ODI mean (SD)	8.2 (15.0)	11.4 (14.2)	16.7 (17.5)	23.7 (20.9)	*p* < 0.05_a_
Back-pain mean (SD)	0.71 (2.83)	1.62 (2.59)	2.69 (2.90)	3.79 (3.40)	*p* < 0.05_a_
Leg-pain mean (SD)	−1.08 (2.75)	1.40 (2.63)	3.53 (2.96)	4.95 (3.41)	*p* < 0.05_a_
EQ -5D mean (SD)	0.12 (0.24)	0.15 (0.29)	0.27 (0.37)	0.38 (0.42)	*p* < 0.05_a_

a = ANOVA-test

The multivariate linear regression analysis (Table 3) shows that the more intense the preoperative leg pain, the greater is the probability of achieving a positive clinical outcome measured as a numerical improvement in ODI-score. This is so even after adjusting for factors like age, smoking, and Body Mass Index and baseline questionnaire-scores (ODI, NRS-score lower extremity pain, NRS-score low back pain and EQ-5D). Groups 3 and 4 had a significantly greater improvement in the primary outcome, compared with group 1. Group 2 also appeared to have a greater improvement compared with group 1, but the difference was not statistically significant. In addition to lower extremity pain, higher age, positive smoking status, higher BMI, high values for preoperative back pain, poor preoperative health condition and low preoperative ODI were factors that were associated with significantly inferior clinical outcomes (see Table 3).

**Table 3:** Results from the multivariate regression analysis. Results from the multivariate linear regression with change in ODI at 12 months after surgery as dependent variable. P-values from a likelihood ratio test, testing whether or not a given variable is important in explaining variations in the data, is reported. The multivariate regression analysis with Group 1 as a reference, shows that the more intense preoperative leg pain the greater is the probability of achieving a positive clinical outcome

Variable	Coefficient (Confidence interval)	*P*-value from LR-test
Preoperative leg pain		
Group 1	Reference	
Group 2	−2.31 (−5.98, 1.36)	
Group 3	−4.65 (−8.25, −1-05)	
Group 4	−6.95 (− 11.28,-2.61)	0.003
Preoperative ODI	−0.58 (−0.65,-0.51)	< 0.001
Age at surgery	0.08 (0.02–0.15)	0.016
Sex		
Male	Reference	
Female	0.83 (−0.63,2.28)	0.265
Smoking		
No	Reference	
Yes	4.76 (3.08,6.44)	< 0.001
BMI	0.24 (0.07,0.41)	0.001
Preoperative back pain		
Group 1	Reference	
Group 2	4.35 (1.13,7.57)	
Group 3	7.11 (3.92,10.30)	
Group 4	8.85 (4.65,13.05)	< 0.001
Preoperative EQ. 5D-score	−1.83 (−4.86,1.20)	0.240
Constant	−6.41 (− 14.85,2.03)	

The percentage of patients classified as being successfully treated (30% better ODI than preoperatively) in each of the four groups is presented in Fig. 4. No differences were found between the four groups (Pearsons Chi-Square), *P* = 0.19. After adjusting for differences in baseline values the predicted number of patients categorized as successfully treated showed significantly difference between group 3 and 4 versus group 1 (*p* = 0.01), but not between group 2 and group 1 (*p* = 0.23).

### Secondary outcomes

**Fig. 3:**
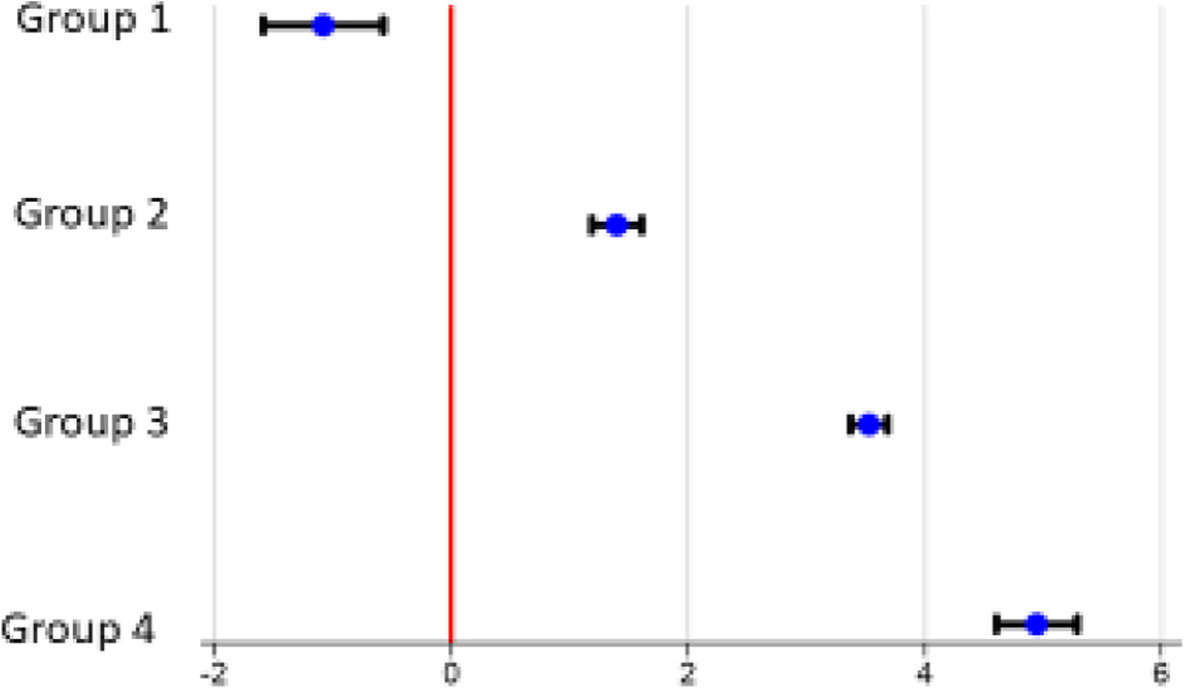
Mean change in lower extremity pain. Mean change in lower extremity pain (with SD) from baseline to 12 months of follow up The patients are divided into four groups according to their preoperative Numeric Rating Scale (NRS)–score for lower extremity pain. Group 1 = NRS-score 0, 1 and 2, group 2 = NRS-score 3, 4 and 5, group 3 = NRS-score 6, 7 and 8 and group 4 = NRS-score 9 and 10 for lower extremity pain

The mean changes from baseline to 12 months follow up are given in Table 2. The trends for the secondary outcome parameters were the same as for the primary outcome, the higher preoperatively NRS-score for leg pain, the greater the probability of a positive clinical outcome after surgery. Group 1 reported a worsening of the mean score for lower extremity pain from baseline to 12 months follow up (Table 3, Fig. 3).

## Discussion

In this cohort we studied patients with lumbar spinal stenosis undergoing decompression surgery, as reported to the Norwegian Registry for Spine Surgery (NORspine). Regarding the primary outcome (change in ODI), and secondary outcomes (change in EQ-5D, and NRS-score for lower extremity pain and back pain), we found a significantly lower improvement among patients with insignificant preoperative lower extremity pain, compared to the other groups with more severe leg pain. These findings are in accordance with the findings of Kleinstuck et al. [26], who reported that more back pain than leg pain at baseline, was associated with a significantly worse outcome after decompression for lumbar spinal stenosis. The multivariate linear regression analysis (Table 3) shows that the more intense the preoperative leg pain, the greater is the probability of achieving a positive clinical outcome, measured as a numerical improvement in the ODI-score.

Patients with insignificant preoperative lower extremity pain reported a worsening in mean values for lower extremity pain after surgery.

**Fig. 4:**
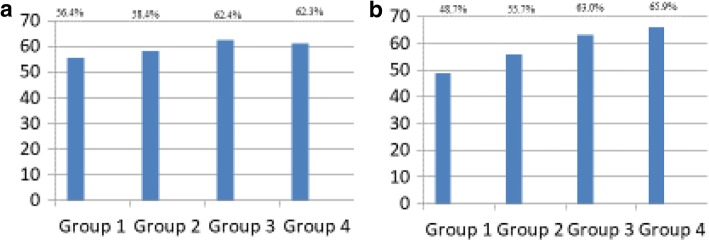
Number of successfully treated patients in the four different groups. **a** Number of successfully treated patients in each group. Success was defined as an improvment from baseline ODI of 30%. No differences between the four groups were found. Pearson Chi-Square test = 4.7755 *P*-value = 0.189. Analysis performed without adjusting for differnces in baseline values. **b** The predicted probility of being classified as successfully treated patient when adjusting for baseline variables The figure show that there are significant differences when comparing group 3 and 4 to group 1, both *p*-values = 0.01. Not significant values when comparing group 2 to group 1, p-value = 0.23. The patients are divided into four groups according to their preoperative Numeric Rating Scale (NRS) –score for lower extremity pain. Group 1 = NRS-score 0, 1 and 2, group 2 = NRS-score 3, 4 and 5, group 3 = NRS-score 6, 7 and 8 and group 4 = NRS-score 9 and 10 for lower extremity pain

However, a majority of patients in the present study reported an improvement in outcome parameters, even those patients with insignificant preoperative lower extremity pain. The mean improvement was 8 ODI points in this group, and 55.6% reported a reduction of at least 30% in ODI score at12 months follow up (Fig. 4a).

It may be argued that patients without pain radiating to the lower extremities do not fulfil the clinical criteria for spinal stenosis and should not have had surgery. Whether the improvement is due to the surgery, or a placebo effect, or the postoperative rehabilitation program, is difficult to determine. It is documented within other orthopedic fields that also sham surgery has effect on clinical outcomes [27–29].

The patients in group 1 had significantly lower pain- and function score at baseline compared to the other groups. They would require less improvement to achieve a 30% reduction of the ODI-score, compared to those high baseline score.. This may be part of the explanation for why the four groups show no significant difference in success rate in the unadjusted analysis. However, when adjusting for the differences in the baseline scores, the logistic regression analysis showed significant difference between the groups in the predicted number of patients reaching a 30% improvement of ODI score. The patients with less lower extremity pain have a statistically significant higher proportion of patients with inferior outcome compared to patients with higher degree of lower extremity pain.

### Limitations of this study

In the NASS-criteria for clinical symptoms it is stated that low back pain, gluteal pain and lower extremity pain are symptoms of lumbar spinal stenosis.

Patients may find it difficult to fit their symptoms into a standardized questionnaire used in a registry, so there may be errors in preoperative classification. It might be difficult to identify the exact location of their symptoms [6]. In the registry forms, patients are asked to quantify their pain intensity (NRS) during the previous week in the lower back or gluteal region, and in the lower extremity. But should they for instance note their pain in the buttock as back pain or leg pain? Furthermore, should buttock pain be registered as radiating pain to the extremity?

Two of the main symptoms of lumbar spinal stenosis, neurogenic claudication and relief of pain while bending forward, are not asked about, either in the patients’ or in the surgeons’ questionnaires. These are amongst factors that may influence the clinical outcome after surgery, which cannot be accounted for in the present register study. Some would claim that patients with insignificant lower extremity pain do not have the cardinal symptom of lumbar spinal stenosis, and therefore should not have undergone surgery. The results of the present study show that the clinical outcome in patients with insignificant lower extremity pain could be interpreted as inferior. Some of these factors may account for the possibly inferior clinical outcome accounted for in patients with insignificant lower extremity pain.

Recently it has been questioned if the Oswestry Disability Index is well enough validated for patients with spinal stenosis (oral presentation in Eurospine convention 2017) [30]. However, a recent publication from the SWESPINE register show a high consistency between a five point Global Assessment-scale and the ODI questionnaire and NRS for low back pain/lower extremity pain in over 94,000 patients, including lumbar spinal stenosis patients [31]. This shows that an improvement of ODI is consistent with the patients’ self-reported effect after surgery.

The success-rate, reported in the present study is based on success criterion of 30% reduction from baseline ODI. There were no differences in the proportion of patients registered as successfully treated in the four groups. This indicates that some patients have benefited from surgery. There is no consensus, to our knowledge, as to what are the best criteria for determining a successful result of surgery for this group of patients, and more research is needed to define optimal criteria for success [32].

The NORspine register does not include objective radiological parameters. The patients and the surgeon can have misinterpreted the symptoms of lumbar spinal stenosis. The Wakayama study from Japan [9], showed that a high proportion of an asymptomatic population had radiological findings corresponding to lumbar spinal stenosis, so the radiological findings of lumbar spinal stenosis may be incidental findings. These factors may also contribute to the inferior results in Group 1.

This is an observational register-cohort study, and therefore reflects a variation of practice amongst the Norwegian surgeons performing spinal surgery. Information about multiple patient-related factors that influence the decision making of the surgeon is not always possible to incorporate in a registry. To answer specific questions, one needs to perform a prospective comparative trial, preferably a randomized trial.

### Strengths of this trial

A register trial has a high external validity. This is a cohort from most of the hospitals performing spinal surgery in Norway in the given period. The high numbers of patients in the study strengthens the validity of the results.

The surgical techniques used in this study are similar, and have been documented not to influence the clinical outcome. All patients in the present study had surgery for lumbar spinal stenosis with spinal decompression with a midline retaining method (unilateral laminotomy with crossover, bilateral laminotomy or spinous process osteotomy), without additional fusion. A previous study from the same register showed similar clinical results after using these three posterior decompression techniques [10].

## Conclusion

In this national register study the analysis show that patients with insignificant lower extremity pain had less improvement in primary and secondary outcome parameters from baseline to follow-up compared to patients with more severe lower extremity pain.

## References

[1] Amundsen T, Weber H, Lilleas F, Nordal HJ, Abdelnoor M, Magnaes B (1995). Lumbar spinal stenosis. Clinical and radiologic features. Spine.

[2] Katz JN, Dalgas M, Stucki G, Katz NP, Bayley J, Fossel AH, Chang LC, Lipson SJ (1995). Degenerative lumbar spinal stenosis. Diagnostic value of the history and physical examination. Arthritis Rheum.

[3] Postacchini F (1999). Surgical management of lumbar spinal stenosis. Spine (Phila Pa 1976).

[4] Kreiner DS, Shaffer WO, Baisden JL, Gilbert TJ, Summers JT, Toton JF, Hwang SW, Mendel RC, Reitman CA, North American Spine S (2013). An evidence-based clinical guideline for the diagnosis and treatment of degenerative lumbar spinal stenosis (update). Spine J.

[5] Tomkins-Lane C, Melloh M, Lurie J, Smuck M, Battie MC, Freeman B, Samartzis D, Hu R, Barz T, Stuber K (2016). ISSLS prize winner: consensus on the clinical diagnosis of lumbar spinal stenosis: results of an international Delphi study. Spine.

[6] Watters WC, Baisden J, Gilbert TJ, Kreiner S, Resnick DK, Bono CM, Ghiselli G, Heggeness MH, Mazanec DJ, O'Neill C (2008). Degenerative lumbar spinal stenosis: an evidence-based clinical guideline for the diagnosis and treatment of degenerative lumbar spinal stenosis. Spine J.

[7] Wai EK, Howse K, Pollock JW, Dornan H, Vexler L, Dagenais S (2009). The reliability of determining "leg dominant pain". Spine J.

[8] Boden SD, Davis DO, Dina TS, Patronas NJ, Wiesel SW (1990). Abnormal magnetic-resonance scans of the lumbar spine in asymptomatic subjects. A prospective investigation. JBone Joint SurgAm.

[9] Ishimoto Y, Yoshimura N, Muraki S, Yamada H, Nagata K, Hashizume H, Takiguchi N, Minamide A, Oka H, Kawaguchi H (2013). Associations between radiographic lumbar spinal stenosis and clinical symptoms in the general population: the Wakayama spine study. OsteoarthritisCartilage.

[10] Hermansen E, Romild UK, Austevoll IM, Solberg T, Storheim K, Brox JI, Hellum C, Indrekvam K. Does surgical technique influence clinical outcome after lumbar spinal stenosis decompression? A comparative effectiveness study from the Norwegian registry for spine surgery. Eur Spine J. 2016. 10.1007/s00586-016-4643-9 27262561

[11] Jones AD, Wafai AM, Easterbrook AL (2014). Improvement in low back pain following spinal decompression: observational study of 119 patients. Eur Spine J.

[12] Nerland US, Jakola AS, Giannadakis C, Solheim O, Weber C, Nygaard OP, Solberg TK, Gulati S (2015). The risk of getting worse: predictors of deterioration after decompressive surgery for lumbar spinal stenosis: a multicenter observational study. World neurosurgery.

[13] Turner JA, Ersek M, Herron L, Deyo R (1992). Surgery for lumbar spinal stenosis. Attempted meta-analysis of the literature. Spine.

[14] Amundsen T, Weber H, Nordal HJ, Magnaes B, Abdelnoor M, Lilleas F (2000). Lumbar spinal stenosis: conservative or surgical management?: a prospective 10-year study. Spine (Phila Pa 1976).

[15] Jacobs WC, Rubinstein SM, Willems PC, Moojen WA, Pellise F, Oner CF, Peul WC, van Tulder MW (2013). The evidence on surgical interventions for low back disorders, an overview of systematic reviews. EurSpine J.

[16] Malmivaara A, Slatis P, Heliovaara M, Sainio P, Kinnunen H, Kankare J, In-Hirvonen N, Seitsalo S, Herno A, Kortekangas P (2007). Surgical or nonoperative treatment for lumbar spinal stenosis? A randomized controlled trial. Spine (Phila Pa 1976).

[17] Weinstein JN, Tosteson TD, Lurie JD, Tosteson A, Blood E, Herkowitz H, Cammisa F, Albert T, Boden SD, Hilibrand A (2010). Surgical versus nonoperative treatment for lumbar spinal stenosis four-year results of the spine patient outcomes research trial. Spine (Phila Pa 1976).

[18] Zaina F, Tomkins-Lane C, Carragee E, Negrini S (2016). Surgical versus nonsurgical treatment for lumbar spinal stenosis. Spine.

[19] Clement RC, Welander A, Stowell C, Cha TD, Chen JL, Davies M, Fairbank JC, Foley KT, Gehrchen M, Hagg O (2015). A proposed set of metrics for standardized outcome reporting in the management of low back pain. Acta Orthop.

[20] Fairbank JC, Pynsent PB (2000). The Oswestry disability index. Spine (Phila Pa 1976).

[21] Ferreira-Valente MA, Pais-Ribeiro JL, Jensen MP. Validity of four pain intensity rating scales. Pain. 2011. 10.1016/j.pain.2011.07.005 21856077

[22] Nord E (1991). EuroQol: health-related quality of life measurement. Valuations of health states by the general public in Norway. Health Policy.

[23] Rabin R, de CF (2001). EQ-5D: a measure of health status from the EuroQol group. AnnMed.

[24] Solberg TK, Olsen JA, Ingebrigtsen T, Hofoss D, Nygaard OP (2005). Health-related quality of life assessment by the EuroQol-5D can provide cost-utility data in the field of low-back surgery. EurSpine J.

[25] Dworkin RH, Turk DC, Wyrwich KW, Beaton D, Cleeland CS, Farrar JT, Haythornthwaite JA, Jensen MP, Kerns RD, Ader DN (2008). Interpreting the clinical importance of treatment outcomes in chronic pain clinical trials: IMMPACT recommendations. J Pain.

[26] Kleinstuck FS, Grob D, Lattig F, Bartanusz V, Porchet F, Jeszenszky D, O'Riordan D, Mannion AF (2009). The influence of preoperative back pain on the outcome of lumbar decompression surgery. Spine (Phila Pa 1976).

[27] Buchbinder R, Osborne RH, Ebeling PR, Wark JD, Mitchell P, Wriedt C, Graves S, Staples MP, Murphy B (2009). A randomized trial of vertebroplasty for painful osteoporotic vertebral fractures. N Engl J Med.

[28] Schroder CP, Skare O, Reikeras O, Mowinckel P, Brox JI. Sham surgery versus labral repair or biceps tenodesis for type II SLAP lesions of the shoulder: a three-armed randomised clinical trial. Br J Sports Med. 2017. 10.1136/bjsports-2016-097098 PMC5754846 28495804

[29] Sihvonen R, Paavola M, Malmivaara A, Itala A, Joukainen A, Nurmi H, Kalske J, Ikonen A, Jarvela T, Jarvinen TA, et al. Arthroscopic partial meniscectomy versus placebo surgery for a degenerative meniscus tear: a 2-year follow-up of the randomised controlled trial. Ann Rheum Dis. 2017. 10.1136/annrheumdis-2017-211172 PMC5867417 28522452

[30] Wertli MMR (2017). Validity of lumbar spinal stenosis outcome measures used in clinical studies: a systematic analysis of randomized and observational clinical trials. Orally presentation in Eurospine convention 2017 Dublin.

[31] Parai C, Hagg O, Lind B, Brisby H. The value of patient global assessment in lumbar spine surgery: an evaluation based on more than 90,000 patients. Eur Spine J. 2017. 10.1007/s00586-017-5331-0 29058135

[32] Copay AG, Martin MM, Subach BR, Carreon LY, Glassman SD, Schuler TC, Berven S (2010). Assessment of spine surgery outcomes: inconsistency of change amongst outcome measurements. Spine J.

